# Taxes on Sugar-Sweetened Beverages to Curb Future Obesity and Diabetes Epidemics

**DOI:** 10.1371/journal.pmed.1001583

**Published:** 2014-01-07

**Authors:** Tony Blakely, Nick Wilson, Bill Kaye-Blake

**Affiliations:** 1 Department of Public Health, University of Otago, Wellington, New Zealand; 2 New Zealand Institute of Economic Research, Wellington, New Zealand

## Abstract

In a linked Perspective, Tony Blakely and colleagues discuss the real-world implications of this type of modeling study.

*Please see later in the article for the Editors' Summary*

**Linked Research Article**

This Perspective discusses the following new study published in *PLOS Medicine*:

Basu S, Vellakkal S, Agrawal S, Stuckler D, Popkin B, et al. (2014) Averting Obesity and Type 2 Diabetes in India through Sugar-Sweetened Beverage Taxation: A Economic-Epidemiologic Modeling Study. PLoS Med 11(1): e1001582. doi:10.1371/journal.pmed.1001582

In this modeling study, Sanjay Basu and colleagues estimate the potential health effects of a sugar-sweetened beverage taxation among various sub-populations in India over the period 2014 to 2023.

## Diet, Sugar-Sweetened Beverages, and Disease Burden

Diet is the leading cause of health loss globally according to the Global Burden of Disease (GBD) 2010, contributing 10% of all health loss [1]. Requiring many assumptions, the GBD team also estimated that 300,000 (95% uncertainty interval: 212,000 to 404,000) deaths per year were attributable to diets high in sugar-sweetened beverages (SSBs) [1], or about 0.6% of all deaths globally per year. Thus the impact of SSBs on global health is not as big as tobacco, alcohol, or salt, but it is still important.

A feature of SSBs is how much they “stand out” as an unnecessary health risk—they tend to have little or no nutritional value, leading to labels such as “empty calories.” Moreover, there are readily available healthy drinks that can be substitutes, such as water, milk, and tea. Low-calorie and diet soda drinks can hardly be considered “healthy” but are also a less hazardous substitute. SSBs are therefore a common target for public health action, from health education to regulation (e.g., bans in schools [2]) to taxes [3].

## Time to Tax Sugar-Sweetened Beverages?

Is it time for countries to consider taxing SSBs or raising existing taxes? This is the topic of the paper by Sanjay Basu and colleagues in this week's *PLOS Medicine*, in which they model the potential impact of a SSB tax for India [4]. Assuming that sales of SSBs continue their non-linear increase, Basu and colleagues estimate that a 20% SSB tax may avert 4.2% of prevalent overweight and obesity, and reduce diabetes incidence by 2.5%, from 2014 to 2023.

Econometric research generally finds that a 1% increase in SSB price should decrease consumption by about 1% [5],[6]. But in real-life settings, such as most states in the US, taxes on SSBs appear to be too small to achieve a measurable impact [7]. Newly introduced SSB taxes, such as the ones in France and Hungary [8], have not yet been evaluated for health impacts in published studies, though reductions in SSB consumption have been reported after such taxes (*Le Figaro* newspaper citation in [6]).

The potential mechanism for SSB taxes to improve population health is clear: taxes increase prices, which decrease consumption and thereby reduce the risk of obesity, diabetes, and other ill effects [9]. For example, Fletcher et al. (2010) estimated that “a one percentage point increase in soft drink taxes decreases adult BMI by 0.003” [9]. Briggs et al. (2013) estimated that in the UK, a 20% tax on SSBs would result in a 1.3 percentage point reduction in obesity, which given that about 25% of the population are obese corresponds to about 5% fewer obese people [6] (and is similar in magnitude to Basu and colleagues' estimate for India [4]).

## Evaluating Disease and Econometric Modeling of Taxes on Food

How much weight should we put on the results of such modeling? Caution is needed, as there are many things to look out for and address with such modeling work [10],[11]: for example, how much consumption will actually change in the future due to price changes—so-called price elasticities; how much consumption of “substitutes” (e.g., fruit drinks and tea in the case at hand) will change—so-called cross-price elasticities, which are even harder to estimate; and future projected disease and risk factor trends *before* considering the tax or subsidy question at hand. There are also the issues of the extent to which manufacturers and retailers pass a tax on to retail prices (tax pass through), and if the tax revenue gets “recycled” by government (e.g., into improved provision of clean reticulated water supplies).

Basu and colleagues, in our view, address the key challenges relatively well. They use Indian price and consumption data to estimate the price elasticities, including for likely substitutes. They also build a baseline or “business as usual” model that projects future disease incidence and future SSB consumption. This latter component is important. A cursory glance at their Table 1 [3] would suggest that SSB consumption is not high enough compared to other substitutes for a “modest” 20% tax to exact the changes estimated in obesity and diabetes. But the future is not the same as the present: SSB consumption is increasing at 13% per year, so it will be a much greater proportion of beverage consumption in the future. Put another way, the effect of SSB consumption on health in ten years' time may be much greater than now due to the projected increased availability of SSBs.

Basu and colleagues also contribute an important new consideration to modeling taxes on SSBs. While they find, consistent with previous research, that consumers do increase net intake of calories from other drinks when they reduce consumption of SSBs, they account for the differences in how the body reacts to those calories via glycemic load. The analysis is more sophisticated than just treating all calories the same.

## Thinking Ahead

The future-orientated aspect of disease and economic decision modeling by Basu and colleagues could be perceived as “brave guess-estimation.” However, astute policy-makers do not just want to know the short-term benefits of an intervention—but also the long-term ones that consider projections into the future (albeit with uncertainty). As concerning as a 13% per annum increase in SSB consumption in India is, it is salient to note that this would still not achieve the high levels of SSB consumption currently experienced in Latin America (see Figure 1a of [9]).

The next step in modeling, but again of great interest to researchers and policy-makers alike, is how interventions play out by sub-populations, or what might be termed heterogeneity or equity effects. With regard to SSBs in India, consumption is currently greater in urban and high-income groups. In the future, that is likely to change with consumption probably becoming higher in rural and low-income groups (as it is in many high-income countries). Model outputs by sub-populations are more uncertain again than the total population.

The world is experiencing massive demographic, epidemiologic, economic, and environmental shifts. There is the epidemic of non-communicable diseases, as well as aging populations, changing levels of poverty and inequality, and strained health sector budgets. Adequately addressing these future challenges will require different policies from today. One potential policy is taxing foods (like SSBs) that produce costs to public health systems and which are not required for nutritional needs. Disease and economic modeling such as that by Basu and colleagues is therefore an important contribution to the evidence base for future-orientated policy making.

## Abbreviations

GBD: Global Burden of Disease
SSBs: sugar-sweetened beverages
